# Similarities and Differences in the Glycosylation Mechanisms in Prokaryotes and Eukaryotes

**DOI:** 10.1155/2010/148178

**Published:** 2011-01-27

**Authors:** Anne Dell, Alaa Galadari, Federico Sastre, Paul Hitchen

**Affiliations:** ^1^Division of Molecular Biosciences and Centre for Integrative Systems Biology, Faculty of Natural Sciences, Imperial College London, London SW7 2AZ, UK; ^2^Faculty of Medicine and Health Sciences, United Arab Emirates University, P.O. BOX 17666, Al-Ain, UAE

## Abstract

Recent years have witnessed a rapid growth in the number and diversity of prokaryotic proteins shown to carry N- and/or O-glycans, with protein glycosylation now considered as fundamental to the biology of these organisms as it is in eukaryotic systems. This article overviews the major glycosylation pathways that are known to exist in eukarya, bacteria and archaea. These are (i) oligosaccharyltransferase (OST)-mediated N-glycosylation which is abundant in eukarya and archaea, but is restricted to a limited range of bacteria; (ii) stepwise cytoplasmic N-glycosylation that has so far only been confirmed in the bacterial domain; (iii) OST-mediated O-glycosylation which appears to be characteristic of bacteria; and (iv) stepwise O-glycosylation which is common in eukarya and bacteria. A key aim of the review is to integrate information from the three domains of life in order to highlight commonalities in glycosylation processes. We show how the OST-mediated N- and O-glycosylation pathways share cytoplasmic assembly of lipid-linked oligosaccharides, flipping across the ER/periplasmic/cytoplasmic membranes, and transferring “*en bloc*” to the protein acceptor. Moreover these hallmarks are mirrored in lipopolysaccharide biosynthesis. Like in eukaryotes, stepwise O-glycosylation occurs on diverse bacterial proteins including flagellins, adhesins, autotransporters and lipoproteins, with O-glycosylation chain extension often coupled with secretory mechanisms.

## 1. Introduction

Protein glycosylation is a phenomenon shared by all domains of life. Over 70% of the eukaryotic proteome is thought to be glycosylated. Although it is too early to predict the full extent of prokaryotic glycosylation, it is clear from the diversity of prokaryotic glycoproteins discovered in recent years that glycosylation in these organisms is the norm rather than the exception. A great deal of progress has been made in understanding prokaryotic glycosylation since the seminal review of Szymanski and Wren in 2005 [1] which focused on the discovery, five years earlier, of a general N-glycosylation system in *Campylobacter jejuni*. The best understood prokaryotic glycoproteins are S-layers, pilins, and flagellins plus a selection of cell surface and secreted proteins which are known to be involved in adhesion and/or biofilm formation. Notably, novel general O-glycosylation systems have recently been uncovered in both pathogenic and symbiotic bacteria. In this paper, our primary aim is to articulate commonalities and differences in eukaryotic and prokaryotic glycosylation rather than provide full coverage of specific areas. There are many excellent specialist reviews referred to throughout our paper which the reader should consult for in depth coverage of particular topics. 

## 2. Oligosaccharyltransferase-Mediated N-Glycosylation Occurs in All Three Domains of Life

### 2.1. Overview

**Figure 1:**
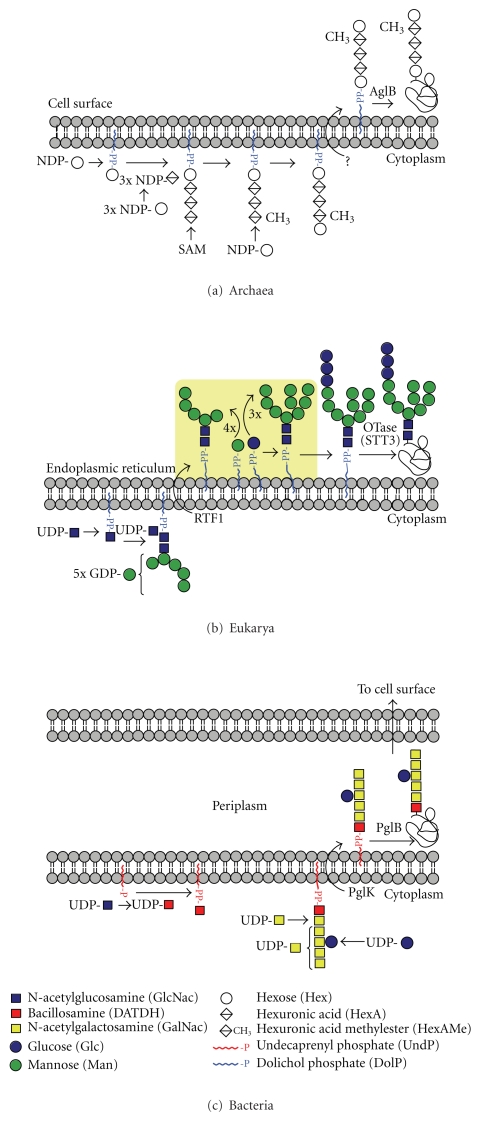
This figure highlights similarities between the biosynthetic pathways of N-linked glycosylation in archaea (a) compared to eukarya (b) and bacteria (c). The elongation steps flagged in yellow in (b) do not have counterparts in (a) and (c).

Until recently it was widely believed that N-glycosylation of proteins is a eukaryotic phenomenon. Nevertheless, it was as long ago as 1976 that Mescher and Strominger reported that the S-layer protein from an archaeal prokaryote, *Halobacterium salinarum*, contained glycans covalently linked to asparagine residues [2]. Over the ensuing three decades sporadic evidence emerged suggesting that N-glycosylation was likely to be common in the S-layers of archaea. In contrast, bacterial S-layers seemed to exclusively carry O-glycans. Then, in the early years of the 21st century, groundbreaking research on the bacterial pathogen *Campylobacter jejuni*, showed that this prokaryote has a general N-glycosylation system [3, 4]. It soon became clear that all three domains of life (Eukarya, Bacteria, and Archaea) perform N-glycosylation in a similar manner. Thus, all engage in stepwise assembly of sugars in the cytoplasm, donated by soluble nucleotide-activated sugars, to form an oligosaccharide precursor attached via pyrophosphate (all domains) or phosphate (archaea) to a lipid carrier (the so-called lipid-linked oligosaccharide or LLO). After assembly of the oligosaccharide, the LLO is flipped from the cytoplasm to face the lumen of the endoplasmic reticulum (ER), or the periplasmic face of the inner membrane, in eukaryotes and Gram-negative bacteria, respectively (Figures 1(b) and 1(c)). Thus far N-glycosylation has not been observed in Gram-positive bacteria. In the case of archaea, which do not have a compartment equivalent to the ER or periplasm, flipping across the cytoplasmic membrane will position the LLO on the exterior surface of the cell where the subsequent transfer to proteins is believed to occur (Figure 1(a)) [5]. In all three cases, the oligosaccharide is subsequently transferred “*en bloc”* from the lipid carrier onto the acceptor protein in a step catalysed by the ubiquitous oligosaccharyltransferase enzyme (N-OST). 

**Figure 2:**
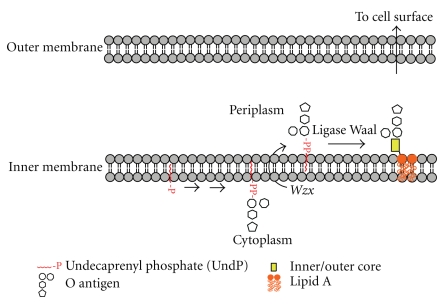
This figure depicts key steps in the LOS and LPS biosynthetic pathway in Gram-negative bacteria which have their parallels in N- and O-glycosylation (Figures 1 and 5). For simplicity, other key steps such as the polymerization of the O-antigen prior to transfer to lipid A involved in LPS biosynthesis are not shown.

Shared and unique aspects of the three hallmark events of N-glycosylation (cytoplasmic assembly of the LLO, flipping across the ER/periplasmic/cytoplasmic membranes, and “*en bloc*” transfer of the oligosaccharide to the protein acceptor) within the three domains are examined in more detail in the next sections. Interestingly these hallmark processes are mirrored in the biosynthesis of bacterial lipo-oligosaccharides (LOS) and lipo-polysaccharides (LPS) [6] (compare Figures 1 and 2). 

### 2.2. LLOs in the Three Domains

**Figure 3:**
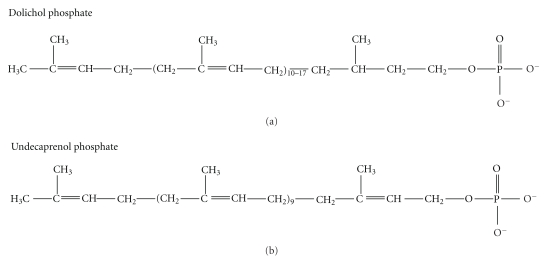
Structures of Dolichol phosphate and Undecaprenol phosphate.

In eukarya and archaea, the lipid constituent of the LLOs is dolichol, which is a polymer of isoprene units (CH_3_–C(CH_3_)=CH–CH_2_–) numbering about 12 in archaea, 14 in yeast, and up to 19 in mammals. Bacteria also have a polyisoprene as their LLO lipid but, instead of dolichol, they use undecaprenol (11 isoprene units) which has one more double bond than the same length dolichol. This double bond is located between carbons 2 and 3 with respect to the alcohol group (see Figure 3). The absence of this specific double bond will confer greater rotational mobility to the oligosaccharidic chain in the dolichol LLOs, compared with the undecaprenol LLOs, which might facilitate chain extension after flipping. 

The LLO biosynthetic pathway has been exhaustively characterized in eukaryotes and is very well understood [7, 8]. Remarkably it is highly conserved in all eukaryotes. Thus the cytoplasmic LLO carries a unique heptasaccharide (Man_5_GlcNAc_2_; Figure 1(b)) which is further elaborated in all higher eukaryotes, after flipping to the lumen of the ER, by the stepwise addition of 4 additional mannoses plus 3 glucoses, donated by dolichol-phosphate-linked sugars, to form Glc_3_Man_9_GlcNAc_2_-P-P-Dol (Figure 1(b)). The glucoses play a pivotal role in lectin-mediated quality control of glycoprotein folding in the ER and are removed by glucosidases during the folding process. In protozoa, however, there is some divergence from the conserved 14 sugar LLO [9, 10]. It has been discovered that these primitive eukaryotes are characterized by LLOs that lack glucose and some are further deficient in the four ER-derived mannoses. This lack of LLO processing in the protist's ER is reminiscent of periplasmic events in bacteria which do not appear to involve the addition of further sugars to their translocated LLOs (see below). 

**Figure 4:**
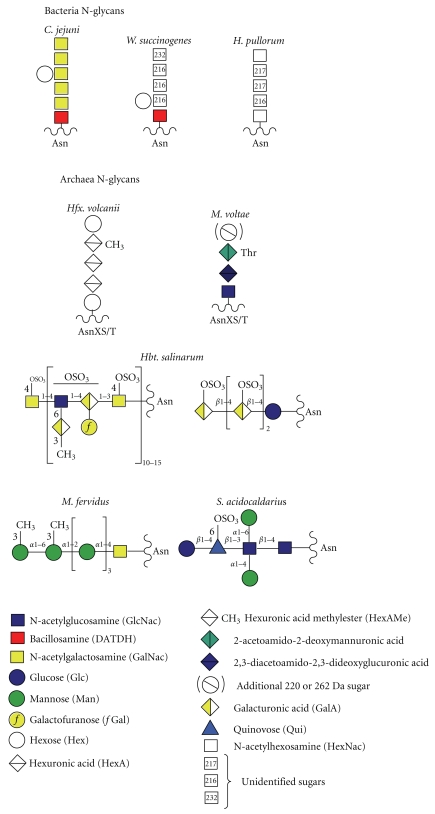
Structures of representative examples of bacterial and archaeal N-glycans.

Although the N-biosynthetic pathways of the three domains have much in common, the archaeal and bacterial LLO processes differ from eukaryotes in two key respects. Firstly, there is no evidence for oligosaccharide sequences being conserved amongst the archaea and bacteria, in contrast to the conserved Glc_3_Man_9_GlcNAc_2_ sequence of all higher eukaryotes. Indeed, as shown in Figure 4, a great diversity of glycans are known to be transferred by N-OSTs to bacterial and archaeal proteins. Despite this diversity, there is some commonality with respect to the type of linking sugar utilized in the three domains. This issue is discussed further in the OST section, below. Secondly, the bacterial and archaeal LLOs do not appear to be further elaborated after flipping. However, it should be borne in mind that knowledge of bacterial and archaeal N-linked glycosylation is only just emerging, and very few biosynthetic pathways have been investigated thus far. Therefore, it remains an open question as to whether LLOs can be extended by stepwise addition of extra sugars in the periplasmic and cell surface compartments. 

### 2.3. Flippases in the Three Domains

Although there exists a very substantial body of evidence, assembled over more than three decades, demonstrating unequivocally that in eukaryotes the LLO precursor is assembled in the cytoplasm and then flipped across the ER membrane to the lumen, remarkably no ER flippase has yet been biochemically identified [11]. Hence comparisons of flippase structures and mechanisms between the three domains are not yet possible. Fortunately, genetic tools have enabled considerable progress to be made in uncovering likely candidates for flippases. Thus, genetic experiments in yeast have provided very good evidence that the RFT1 protein is involved in transfer of the Man_5_GlcNAc_2_-LLO across the ER membrane [12]. In accordance with the conclusion that they play a role in translocation, RFT1 proteins are conserved in eukaryotic organisms, although it is still not clear whether they are actually the elusive flippases [11].

The elucidation of archaeal N-biosynthetic pathways is still in its infancy (see Calo et al. [5] for an in-depth review of recent discoveries) and thus far flippases have not been studied in this domain. In contrast, bacterial flippases are quite well understood, not the least because LLO translocation is integral to LPS biosynthetic pathways which have been intensively studied for many years. It is known, for example, that the product of the *Wzx* gene, a non-ABC-type transporter, mediates transport of undecaprenol-linked O-antigen subunits across the plasma membrane in LPS biosynthesis [13]. With respect to the bacterial N-glycosylation pathway, which has been rigorously studied in the “paradigm” organism, *C. jejuni*, Aebi, and coworkers have shown that PglK (previously called wlaB), which is an ABC-type transporter, is responsible for flipping the LLO [14]. Interestingly, these workers found that PglK has a relaxed substrate specificity exemplified by its ability to complement a Wzx deficiency in O-antigen biosynthesis in *E. coli*. 

Notably, all bacterial N-glycans identified to date have seven or fewer sugar residues, with many archaeal structures being of a similar size (Figure 4). As described earlier, the eukaryal cytoplasmic LLO contains seven sugars (see Figure 1(b)). These observations suggest that a maximum of seven sugars might be optimal for the flipping mechanism, though it has also been suggested that the flipping process might be affected by monosaccharide composition at the reducing end of the glycan [14]. In this context, it could be significant that the large archaeal N-linked polysaccharide shown in Figure 4 is composed of tandem repeats of a short oligosaccharide. This type of structure is reminiscent of bacterial LPS and could therefore be assembled from short LLO precursors, after flipping across the cytoplasmic membrane, in a similar way to Wzx/Wzy-dependent O-antigen polymerization in the periplasm of bacteria [13]. Alternatively, it possible that this N-linked polysaccharide might be flipped across the membrane in an ATP-binding cassette (ABC) transporter-dependent manor [15].

### 2.4. N-OSTs in the Three Domains

The transfer of oligosaccharides from the LLOs to asparagine acceptors in N-linked glycoproteins is catalysed by homologous oligosaccharyltransferase enzymes (N-OSTs) in the three domains of life. In eukaryotes and archaea; N-OSTs are ubiquitous. Consequently, N-linked glycoproteins are found in abundance throughout both domains. On the other hand, bacteria have probably not evolved N-OSTs of their own (see below) and N-glycosylation is restricted to a limited number of species. The first bacterial N-OST gene was identified about a decade ago. This is the *p*gl*B* gene of *Campylobacter jejuni* which was found to be highly homologous to the catalytic subunit (called Stt3) of eukaryotic N-OSTs. A similar degree of homology was found in archaeal N-OST genes (which are called *aglB*) when their identity was confirmed a few years later [16, 17]. 

When the general N-glycosylation system was first discovered in *C. jejuni, * it was thought to be unique, and it was postulated that this organism might have acquired the *pglB* gene by lateral gene transfer from either the archaeal or the eukaryal domains [4]. It is now considered most likely that *pglB* originated from archaea rather than eukarya (Brendan Wren, London School of Hygiene and Tropical Medicine, personal communication). This conclusion is based on knowledge emerging from searches of bacterial genomes for *pglB* orthologues. Thus far, bacterial N-OST candidates have been found exclusively in a subset of species belonging to the phylogenetic grouping known as the epsilon subdivision of the Proteobacteria, which include *Campylobacter*, *Helicobacter,* and *Wolinella* genera. Amongst these, N-glycosylation has been rigorously confirmed by mass spectrometry for *C. jejuni*, *W. succinogenes,* and *H. pullorum* (Figure 4) [1, 18, 19]. Note, however, that although *H. pullorum* has the machinery for N-glycosylation, the *pglB* gene is absent in related mammalian pathogens such as *H. pylori* and *H. hepaticus*. It may be significant that in primordial deep sea vents, which are the homes for many archaea, the majority of bacteria are epsilon proteobacteria. So it is tempting to speculate that these extreme environments have provided the conditions for N-OST gene transfer between the prokaryotic domains (Brendan Wren, personal communication). 

The preceding section has focused on the genes encoding the N-OST enzymes. We now overview current understanding of the biochemistry of N-OSTs across the three domains of life. N-OSTs in archaea, bacteria, and primitive eukaryotes (protozoa) are comprised of a single subunit (the catalytic subunit) which is the product of the aforementioned *aglB*, *pglB, * and *Stt3* genes, respectively. In contrast, all N-OSTs of higher eukaryotes are multi-subunit complexes in which the catalytic subunit (Stt3) is accompanied by a total of seven additional proteins whose roles remain poorly understood [14, 20–22]. Suggested functions of these accessory proteins include regulating substrate specificity, possibly by expanding the range of acceptor sequences, and assisting in protein translocation and/or folding. Why primitive eukaryotes do not require a multiprotein complex remains enigmatic, but even more enigmatic is the observation that a single Stt3 from *Leishmania major* can substitute for the whole N-OST complex in yeast [23, 24]. 

It is quite normal for eukaryotes to have more than one *Stt3* gene. The highest number has been found in primitive eukaryotes. For example, *L. major* expresses four Stt3 paralogs, whilst *Trypanosoma brucei* has three. The latter has been shown to have distinct LLO and glycosylation site preferences [10]. Yeast, however, has only a single *Stt3* gene (called *Stt3p*), whilst vertebrates, insects, and plants have two, encoding for Stt3A and Stt3B, respectively. It has been shown, via siRNA knockdown experiments in mammalian cells, that Stt3A glycosylates cotranslationally, whilst Stt3B, which is normally coexpressed, acts posttranslationally, although the protein must not be folded. Also, Stt3B is required for efficient glycosylation adjacent to the N-terminal signal sequence [25]. Thus the two isoforms appear to function in concert to ensure maximal efficiency of N-glycosylation. 

Information is only just beginning to emerge concerning the number of N-OST genes in prokaryotes. *Campylobacter* has only a single *pglB* gene but *H. pullorum* has two unrelated genes, denoted *pglB1* and *pglB2*, the first of which has been proven to mediate glycosylation [18]. The role of the pglB2 protein is not known. Bioinformatic searches for *aglB* genes in archaea have suggested multiple candidates in individual organisms but confirmation of expression and activity has not yet been determined experimentally [5].

N-OSTs from all domains have been found to exhibit quite relaxed specificity with respect to the oligosaccharide donor. Thus each is capable of transferring short glycans from biosynthetic LLO intermediates in addition to the full length glycans of the mature LLOs. Eukaryotic and bacterial N-OSTs transfer glycans whose reducing sugar carries at least one acetamido (NAc) group. Thus the eukaryotic N-glycan has a chitobiose core (GlcNAcb1-4GlcNAc) and the linking sugar in characterized bacterial N-glycans is either 2,4-diacetamido-2,4,6-trideoxyglucopyranose (bacillosamine; *Campylobacter* and *Wolinella*) or HexNAc (*H. pullorum*). Interestingly, the pglB protein of *C. jejuni* is capable of transferring a variety of O-antigen oligosaccharides onto protein acceptors in engineered *E. coli* cells, provided their reducing sugar has an NAc moiety [26]. This discovery has important implications for the development of O-antigen containing neoglycoprotein vaccines. Archaea appear to have a greater diversity of linking sugars, including Glc as well as GlcNAc and GalNAc [5, 17]. Interestingly, a *Sulfolobus* archaeal species, which is very close phylogenetically to primitive eukaryotes, has mannose rich chitobiose-linked N-glycans reminiscent of the eukaryotic core sequence [27]. 

When comparing mechanisms of prokaryotic and eukaryotic N-glycosylation, it is important to remember that the folding status of their proteins is very different at the time of glycosylation. Thus, eukaryotic oligosaccharides are transferred to nascent proteins before they are folded, whilst in prokaryotes the proteins are presumably fully folded, having already been transported from the cytoplasm, where translation occurs, into the periplasm or onto the surface, where glycosylation takes place. In all three domains, the asparagine acceptor must normally be located in a consensus sequence (Asn-X-Ser/Thr or, rarely, Asn-X-Cys, where X cannot be proline); however, not all consensus sequences are glycosylated. Sequence motifs contributing to specificity of site occupancy are not yet fully understood, but it is already clear that bacterial glycosylation is much more restricted than eukaryotic glycosylation. For example, consensus sites in *C. jejuni* require an upstream Glu or Asp residue in the extended consensus sequence D/EZNXS/T, where neither Z nor X can be proline [28]. High throughput glycoproteomic efforts are beginning to provide comprehensive site-occupancy data in eukaryotic systems [29]. It is hoped that these and similar experiments will facilitate the development of algorithms that will be capable of accurately predicting which consensus sequences in eukaryotic proteomes are likely to be occupied. 

In contrast to eukaryotes, very few prokaryotic glycoproteins have had their glycosylation sites determined. Based on a limited body of data, some predictions have been made for sequences favouring archaeal glycosylation [5] but emerging data from studies of Sulfolobus S-layers suggest that these rules probably will not be universally applicable (see [27] and unpublished work from our laboratory). Bearing in mind that glycosylation in prokaryotes occurs posttranslationally, it is conceivable that general rules for site occupancy may not prevail in these organisms, because of the unique nature of individual proteins with respect to accessible consensus sequences. This could be especially relevant in S-layer glycosylation, because these proteins self-assemble into crystalline monolayers and all their consensus sequences are therefore likely to be in exposed locations [30, 31].

In concluding this section on N-OSTs, we draw attention to the fact that crystal structures are now available for the C-terminal domains, that include the WWDYG motif implicated in the catalytic mechanism, of both an archaeal AglB and the PglB of *C. jejuni*, although not so far for the eukaryotic Stt3, despite many valiant efforts [20]. Mechanistic and evolutionary understanding provided by the crystal structures has been reviewed very recently [5] so will not be covered here.

### 2.5. Glycoprotein Remodelling

All eukaryotic glycoproteins are subjected to extensive remodelling in the Golgi apparatus after they exit the ER, resulting in heterogeneous mixtures of glycoforms exhibiting a great variety of peripheral structures, many of which are rich in functionally important sugars such as fucose and sialic acid [7, 8]. Prokaryotes have no counterpart to the Golgi apparatus, and there is no evidence so far that they remodel their N-linked glycoproteins.

## 3. *Haemophilus influenzae* Can Perform Cytoplasmic N-Glycosylation

About seven years ago a study of the HMW1 adhesin of *H. influenzae* uncovered a potentially novel N-glycosylation pathway occurring in the cytoplasm of this bacterium [32]. This intriguing discovery has now been confirmed by rigorous structure analyses which, remarkably, have identified 31 glycosylated Asn residues within the HMW1 protein [33, 34]. All sites carry either Hex or Hex-Hex, where Hex can be Gal or Glc, and all but one of the glycosylation sites has the normal N-glycosylation consensus sequence (Asn-X-Ser/Thr, see Section 2). The cytoplasmic enzyme responsible for glycosylation has been confirmed as HMW1C. Interestingly it transfers glucose to all glycosylated asparagines but only transfers galactose to a subset of these sites. Moreover, the same enzyme appears to be responsible for the Hex-Hex glycoforms as well as those carrying a single Glc or Gal. The mechanisms of these processes remain to be established. Homology analysis suggests that a variety of other bacteria possess HMW1C-like proteins, so it is likely that this type of cytoplasmic N-glycosylation will be found elsewhere. Whether similar glycosylation occurs in archaea is not known. As shown in Figure 4, glucose has been observed as a linking sugar in some archaeal N-glycans, but it is likely that the N-OST pathway is employed in their biosynthesis [5]. The presence of a Glc-Asn moiety was reported in eukaryotic laminin in 1994 [35] but this observation has not been independently confirmed.

## 4. Bacteria Have Oligosaccharyltransferase-Mediated O-Glycosylation Pathways

### 4.1. Overview

**Figure 5:**
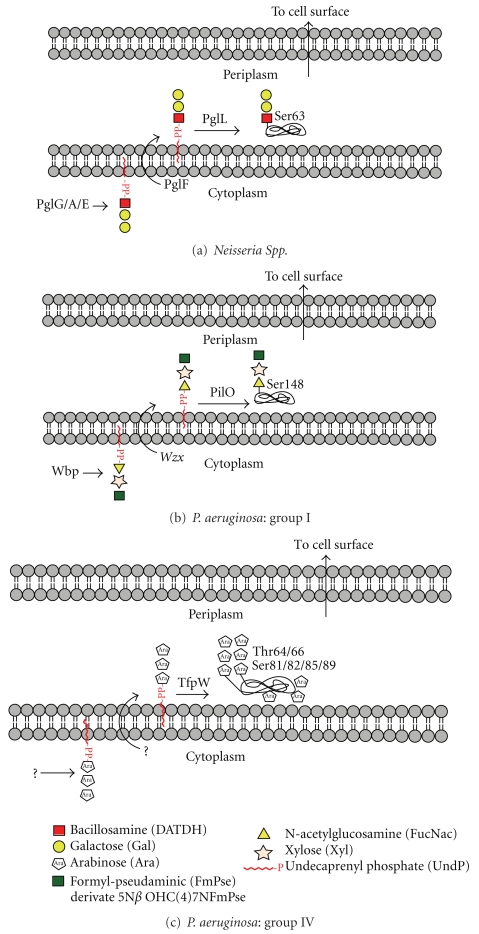
This figure shows key steps in the O-oligosaccharyltransferase-mediated O-glycosylation pathways in *Neisseria *and *Pseudomonas. *Note the similarities to N-glycosylation (Figure 1) and LPS biosynthesis (Figure 2).

During the past five years, intensive research on *Neisseria* and *Pseudomonas* pilin glycosylation has uncovered a general O-glycosylation pathway that, remarkably, has all the hallmarks of N-linked glycosylation (Figure 5). Moreover, this general pathway does not appear to be restricted to a few pilin proteins in a handful of pathogens. Thus, very interesting data are emerging from research on *Bacteroides* species that suggests that these bacteria are capable of glycosylating a great number of proteins in this way. So far oligosaccharyltransferase-mediated O-glycosylation has only been found in Gram-negative bacteria, which is perhaps not surprising, bearing in mind that it mirrors LPS biosynthesis. 

### 4.2. Neisseria and Pseudomonas Pilins

Much of our knowledge of bacterial O-linked glycosylation pathways has been elucidated from studies in Neisseria species. O-linked glycosylation was first characterised in* Neisseria meningitidis*, where the pilin protein was shown to be modified by a trisaccharide [36], with a similar glycan being found on *N. gonorrhoeae * [37]. Subsequent bioinformatics and directed mutagenesis led to the identification of an O-OST, called PglL, in *N gonorrhoeae* [38, 39]. PglL O-OSTs belong to a family of bacterial OSTs responsible for O-linked glycosylation of type IV pilins. This family appears to be widespread amongst pathogenic bacteria, including some strains of *Pseudomonas aeruginosa*, where it is called PilO [40, 41]. Moreover, it has recently been demonstrated that Neisseria are able to decorate a diverse set of proteins via the O-OST pathway [42, 43]. 

Research using Neisseria and Pseudomonas glycosylation systems in engineered* E. coli* cells has demonstrated that the biosynthesis of the O-linked glycan has a number of similarities to its N-linked counterpart (compare Figures 1 and 5). The O-linked glycosylation pathway involves LLOs, and the glycans are transferred *en bloc* by the O-OSTs from the LLOs carrier onto the protein [39]. The translocation of the LLO substrate into the periplasm is required for activity and it has been shown that PglF, a protein with homology to O-antigen “flippase,” is required for pilin glycosylation which is thought to occur in an analogous manner to the Wzy-dependent addition of O-antigen to the core-LPS [38]. In a similar fashion to the N-OSTs in archaea, bacteria, and lower eukaryotes, the O-OST's catalytic subunit is sufficient for glycosylation. As with O-glycosylation in eukaryotes, there appears to be no consensus sequence for defining sites of O-glycan attachment. Interestingly, the Neisseria O-OSTs display a pronounced substrate promiscuity when compared to N-OSTs, as demonstrated by their ability to transfer virtually any glycan from an LLO carrier onto pilin in engineered *E. coli* cells. For example, it was shown that the Neisseria PglL could transfer peptidoglycan subunits onto pilin, highlighting the potential for exploitation of such pathways for biotechnological purposes [44]. As such it appears that the substrate specificity of the O-OSTs is found in the lipid carrier, a hypothesis nicely demonstrated using an *in vitro* glycosylation system that utilised purified Neisseria PglL, pilin, and the lipid farnesyl pyrophosphate carrying a synthetic pentasaccharide that was successfully transferred onto the pilin protein [44].

### 4.3. Bacteroides Species Have an OST-Mediated O-Glycosylation Pathway

Bacteroides comprise one of the most abundant genera of commensals in the human colon. Exciting recent research suggests that these bacteria are not only capable of O-glycosylating many of their proteins but, unusually, they exploit a host-like pathway to add fucose (apparently acquired from their host glycans and/or from plant polysaccharides present in the gut) onto their glycoproteins and polysaccharides [45]. A combination of cell biology and molecular biology experiments has provided convincing evidence for the existence of a general O-glycosylation system in these symbiotic bacteria which has all the hallmarks of the pilin O-OST-mediated pathogen pathway described earlier [46]. Notably, O-glycosylation appears to be central to the physiology of *B. fragilis* as well as its ability to colonise its ecological niche. Although the structures of the *B. fragilis* O-glycans remain to be defined, many elements of the biosynthetic pathway are beginning to be unraveled. Thus five glycosyltransferases, plus an unrelated fucosyltransferase, have been proposed to be involved in assembly of the LLO on the cytoplasmic face of the inner membrane. Translocation to the periplasm is thought to be mediated by the O-antigen flippase (Wzx, see Figure 2). However, there is no candidate gene as yet for the putative O-OST. 

Interestingly, very recently it has been reported that fucosylated O-glycans are present on the fimbriae of *Porphyromonas gingivalis *[47]. Like *B. fragilis*, this oral mucosal pathogen is a member of the Bacteroides genus. It is conceivable, therefore, that *P. gingivalis* glycosylates its proteins via a similar pathway to *B. fragilis*. Monosaccharide compositional analysis has shown that the *P. gingivalis* glycans are likely to be complex (Fuc, Xyl, Man, Gal, Glc, GalNAc and GlcNAc have all been detected) but so far no sequences are available for this glycoprotein.

## 5. Processive O-Glycosylation Systems in Eukaryotes and Prokaryotes

### 5.1. Overview

All eukaryotic O-glycosylation is processive that is, it is a stepwise process which begins with the attachment of the linking monosaccharide to the acceptor serine or threonine. Further sugars are added one at a time to form the mature glycan. Many eukaryotic O-glycans are of the mucin-type which are linked via GalNAc, but other classes exist which are attached to proteins via a variety of sugars including Fuc, Man, Glc, Gal, Ara, Xyl, and GlcNAc. Most eukaryotic O-biosynthetic events take place in the Golgi, although some classes of O-glycans, for example, O-Man linked glycans (see later), are initiated in the ER. An enormous variety of sequences can be attached to these linking sugars. Thus there is a great diversity of O-glycosylation in the eukaryotic domain [6, 7]. 

Archaeal O-glycosylation has rarely been investigated. The only substantive study was about twenty years ago when the S-layers of *Halobacterium salinarum* and *Haloferax volcanii* were shown to carry several O-linked disaccharides of sequence Glc1-2Gal [48]. In contrast, there is a large body of evidence pointing to a rich diversity of O-glycans in the bacterial domain. The most complex structures have been found on bacterial S-layers which have been investigated in many species of bacteria over the last thirty years. Their structures and biosynthesis have been comprehensively reviewed on several occasions [5, 31, 49, 50] and the reader is referred to these articles for further information. Below we discuss emerging understanding of other families of bacterial cell surface and secreted O-linked glycoproteins, many of which, in contrast to the S-layers, have structural and/or functional counterparts in the eukaryotic domain.

### 5.2. Mucin-Like Glycoproteins

Mucins are high molecular weight eukaryotic glycoproteins, produced in abundance by epithelial and goblet cells, whose polypeptide chains are coded by the MUC genes [7]. Mucins are characterized by the presence of tandem repeats of serine/threonine/proline-rich sequences which are extensively O-glycosylated. Mucins readily form gels and are a key component of most gel-like secretions in eukaryotes where they have functions ranging from lubrication to serving as receptors for microbes. In recent years, it has become evident that many bacterial biofilms contain glycoproteins whose compositions indicate that they are mucin-like molecules [51–53]. The best characterized are the serine-rich repeat (SRR) glycoproteins belonging to the Fap1 family, which are conserved in Streptococci, Staphylococci, and Lactobacilli, and are required for bacterial biofilm formation and pathogenesis. 

**Figure 6:**
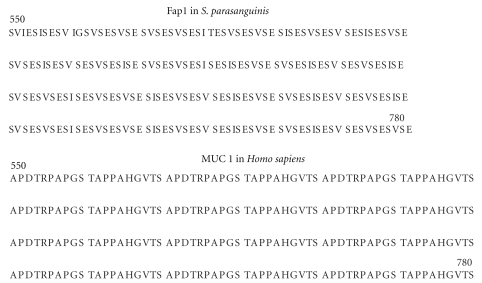
Comparison of mucin-like sequences in bacteria with mammalian mucins. Partial sequences of Fap1 in *S. parasanguinis *and MUC 1 in *Homo sapiens* are shown.

The polypeptides of SRR family members are comprised of a long signal peptide followed in turn by a short serine-rich domain, an acidic or basic region, a long serine-rich domain and a C-terminal anchoring motif. The SRR sequences are reminiscent of the eukaryotic mucins in that the majority of the polypeptide is comprised of tandem repeats of short motifs which have related sequences (see Figure 6 for comparison of a portion of an SRR domain compared with part of the human MUC-1 sequence). The serine-rich domains have the key hallmark of the mucins, namely, variable repeated sequences rich in potential O-glycosylation sites (see Figure 6), but in contrast to the mammalian mucins, proline is absent from the SRR domains. Eukaryotic mucin glycosylation is initiated in the Golgi after the protein is fully folded. Thus their proline residues are important for ensuring that exposed, accessible sites are available for glycosylation. Currently little is known about the process of glycan attachment to the bacterial SRRs, other than the fact that attachment of the linking sugar appears to occur very rapidly in the cytoplasm, before transport to the cell surface via the accessory Sec transporter. It has been proposed that the glycosylation mechanism is a two-step process, with the second step requiring several accessory secretion components and thus is probably coupled with secretion [53]. Recent electron microscopy structural studies have indicated that the serine dipeptide repeat domains have a super-helical extended structure with exposed serine side-chains, which are expected to be readily accessible to O-glycosylation [54]. 

The glycan content of the SRRs has been explored in five species, *Streptococcus parasanguinis*, *S gordonii [55], S. agalactiae [56], S. pneumoniae [57], *and *Staphylococcal aureus*. This has been largely done using lectins such as wheat germ agglutinin (WGA) which recognize terminal GlcNAc, and by sugar compositional analyses [53]. No complete glycan structures have been defined so far. Lectin blotting, supplemented by sugar composition data, has indicated that the linking sugar in the SRRs is probably GlcNAc. Interestingly two glycosyltransferases, called Gtf1 and Gtf2, are required for this initial glycosylation step by *S. parasanguinis* [58]. Gtf1 and Gtf2 homologs from *S. pneumoniae* also form an enzyme complex that catalyzes the transfer of GlcNAc to serine-rich sites of PsrP [59]. A similar requirement for two glycosyltransferases adding a single sugar occurs in the O-mannosyl glycans of higher eukaryotes (see later) whose biosynthesis is initiated by a heterodimer enzyme complex composed of protein O-mannosyltransferases (POMT) 1 and 2 [7]. It should be noted that although O-linked GlcNAc is found on eukaryotic cytoplasmic and nuclear proteins, it is probably not analogous to SRR O-glycosylation, because eukaryotic O-linked GlcNAc residues are not further extended. Moreover, this ubiquitous eukaryotic glycosylation is unusual because it is dynamic and involves cross-talk with phosphorylation [60]. 

A glucosyltransferase has recently been identified in *S. parasanguinis* that transfers glucose to the GlcNAc-modified Fap1 [61]. Although the structure of the product of glycosylation remains to be determined, it is tempting to speculate that these bacterial proteins might carry glycans whose core sequences are glucosyl analogues of the core type 1 sequence, Gal*β*1-3GalNAc, that is ubiquitous in mammalian mucins. Sugars additional to Glc and GlcNAc, including GalNAc and Rha, have been observed at low levels in sugar analyses of the Fap1 glycoproteins [53]. It remains to be established whether the putative Glc-GlcNAc moieties are further elongated or whether other glycans account for the compositional data.

### 5.3. O-Linked Mannose Glycosylation

The title of a recent review “*Protein O-mannosylation: conserved from bacteria to humans*” [62] encapsulates the importance of this class of glycosylation. In eukaryotes, O-mannosyl glycans are abundant in yeast and fungi, whilst in mammals they occur on a restricted number of proteins, such as *α*-dystroglycan where their impairment is a cause of congenital muscular dystrophy [63, 64]. Yeast and fungi express short mannosyl oligomers, with galactose being present on terminal sites in some species. In contrast, mammalian O-mannosyl glycans carry sialylated and fucosylated N-acetyllactosamine sequences similar to those found in mucins. Eukaryotic O-mannosylation is initiated in the ER by the concerted action of two protein O-mannosyltransferases (POMT1 and 2) which employ Dol-P-Man as the mannose donor. Chain extension subsequently takes place in the Golgi. The review cited above gives a comprehensive account of these events.

O-mannosyl glycans analogous to those found in yeast and fungi have been found on glycoproteins from members of the Actinomycetes class of Gram-positive bacteria which include the Mycobacteria and Streptomyces genera [62, 65]. The first to be characterised were the surface glycoproteins of *Mycobacterium tuberculosis * [66, 67]. Subsequently *M. bovis* [68], *Corynebacterium glutamicum* [69], and *Streptomyces coelicolor* [70] were shown to be similarly glycosylated. All contain O-glycans whose sequences are restricted to short stretches of mannose (usually three residues or less). Like in eukaryotes, the mannosyl donor is a polyprenol phosphate, and their protein O-mannosyltransferases (POMTs) are membrane associated. The activities of the products of candidate POMT genes in *M. tuberculosis*, *C. glutamicum,* and *S. coelicolor* have been genetically and biochemically confirmed. In contrast to eukaryotes, heterodimeric enzymes do not appear to be required. The fact that bioinformatic screening has uncovered a plethora of POMT homologs in other species, indicates that O-mannosylation constitutes a general O-glycosylation pathway in Actinomycetes. 

Steps equivalent to the eukaryotic Golgi processes of O-mannose extension have not been determined thus far in bacteria. There are, nevertheless, candidate mannosyltransferases, for example, those involved in the biosynthesis of the mannan core of cell wall lipomannan/lipoarabinomannan in Mycobacteria [62]. It has been suggested that O-mannose extension in Mycobacteria is coupled with Sec-dependent secretion in a manner akin to that proposed for the serine-rich proteins in Streptococci and Staphylococci described earlier [53]. 

### 5.4. O-Linked Heptose in *E. coli*

**Figure 7:**
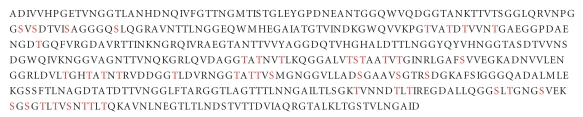
Sequence of the passenger domain of the autotransporter protein Ag43 from *E. coli *with heptosylation sites indicated in red.

Autotransporters constitute the biggest group of secreted proteins in Gram-negative bacteria. AIDA-I, TibA, and Ag43 are three autotransporter proteins in pathogenic *E. coli* which are associated with virulence phenotypes, such as the formation of biofilms and aggregates. All three are extensively glycosylated with O-linked heptose on their so-called “passenger domains” [71–74]. Glycosylation occurs in the cytoplasm and the heptoses are derived from ADP-*glycero*-*manno*-heptopyranose which is recruited from the LPS biosynthetic pathway. The passenger domains are secreted to the extracellular environment where their glycosylation appears to enhance bacterial attachment to human cells. The heptoses are attached at multiple sites in Ser/Thr rich domains (Figure 7) that are reminiscent of eukaryotic mucin sequences (see earlier) although they lack the hallmark tandem repeats of the latter. 

### 5.5. O-Glycosylation of Bacterial Flagellins

Flagellin O-linked glycosylation has been widely reported in a number of bacteria, where it appears to be restricted, with the archaeal counterparts being N-linked. Current knowledge of the O-linked sugars involved in flagellin glycosylation has been covered in recent reviews [75, 76]. Probably the best studied flagellin glycans are found to be glycosylated with a family of “sialic acid-like” monosaccharides, based around the sugars pseudaminic acid and legionaminic acid, with a diversity being generated by variation in their decorating appendages. These sugars have been found in several species including *Campylobacter jejuni*, *Helicobacter pylori,* and *Aeromonas caviae*. In *C. jejuni*, the O-linked glycosylation gene cluster has been identified and the function of a number of gene products involved in the pseudaminic acid biosynthetic pathway has been elucidated. It appears to have evolved to share some of the same biosynthetic machinery as the N-linked glycosylation pathway, allowing the organism to maintain a compact genome and avoid redundancy [1]. Flagellin glycosylation in *P aeruginosa* has also been shown to share biosynthetic machinery, in this case with the O-antigen pathway [77].

The most complex flagellin O-glycans identified thus far have been found in hypervirulent strains of *Clostridium difficile* [75, 78]. The *C. difficile* flagellins carry HexNAc-linked oligosaccharides up to at least five sugars in length.

 In contrast to the “*en bloc*” transfer in the N-linked pathway, it is apparent that flagellin O-linked glycosylation is likely to proceed in a sequential fashion. Given that a single sugar residue is often added to sites of attachment, specific glycosyltransferases are thought to be involved in the glycosylation process, but our present understanding of the glycosyltransferases involved in the glycosylation process remains limited. The current proposed model for O-linked flagellin glycosylation occurs at the cytoplasmic face of the inner membrane in the vicinity of the type III secretion complex [76]. Nucleotide activated sugars are utilised by specific glycosyltransferases in the glycosylation machinery and are added to exposed serine and threonine residues. The glycosylated flagellin monomers are then secreted to the tip of the growing flagellin filament. 

## 6. Predicting O-Glycosylation Sites in Bacterial Proteins

Unlike N-glycosylation, there is no consensus sequence for O-glycosylation. However, it is well known that certain sequence motifs are preferred in mucin type O-glycosylation. Indeed the NetOglyc open access tool, which can be very helpful for predicting possible sites of eukaryotic mucin O-glycosylation, was developed using knowledge of preferred sequence motifs [79]. The rapid progress that is being made in defining O-glycosylation sites in diverse prokaryotic glycoproteins, coupled with the fact that some researchers are beginning to employ the NetOglyc tool to guide them in the choice of targets for mutation in searches for prokaryotic glycosylation [67, 80], have made it timely to assess the applicability of the NetOglyc tool to prokaryotic glycoprotein research. 

In a preliminary unpublished study, we have ascertained NetOglyc predictions for selected members of each of the families of prokaryotic O-glycoproteins described in the previous sections, for which there is published experimental data on site occupancy. The outputs send the very clear message that NetOglyc does not, in fact, correctly predict O-glycosylation in most families of prokaryotic glycoproteins. Thus we found that no sites were correctly predicted in the pilins, flagellins, serine-rich proteins, the autotransporters, or the BF2494 glycoprotein of *B. fragilis*. Experimental data [46] suggests that the latter has an essential three-residue glycosylation site motif (D)(S/T)(A/I/L/V), which is not typical of eukaryotes, so it is therefore not surprising that NetOglyc appears to be not suitable for predicting this type of O-glycosylation. In conclusion, NetOglyc is likely to only be useful for predicting O-glycosylation in prokaryotic glycoproteins like mycobacterial lipoproteins whose Pro/Ser/Thr-rich glycosylation domains have sequence characteristics which they share with the mammalian mucins [67]. Prediction in other families will require the development of new algorithms which take account of their specific glycosylation domain characteristics.

## 7. Conclusions

Although much remains to be uncovered concerning protein glycosylation in prokaryotes, several themes are emerging from the discoveries of the past decade. Firstly, like in eukaryotes, N-glycosylation is largely restricted to Asn-X-Ser/Thr consensus sequences, even when the canonical oligosaccharyltransferase pathway is not involved. Secondly O-glycosylation is far more abundant in bacteria than in archaea whilst the reverse is true for N-glycosylation. Thirdly, oligosaccharyltransferase-mediated O-glycosylation is likely to be widespread in Gram-negative bacteria. In contrast this type of O-glycosylation has not thus far been proven to occur experimentally in Gram-positive bacteria or archaea. Finally cytoplasmic O-glycosylation appears to be both more common and more diverse in Gram-positive compared with Gram-negative bacteria, possibly because of the existence of the alternative periplasmic O-OST pathway of the latter.

## References

[1] Szymanski CM, Wren BW (2005). Protein glycosylation in bacterial mucosal pathogens. *Nature Reviews Microbiology*.

[2] Mescher MF, Strominger JL (1976). Purification and characterization of a prokaryotic glycoprotein from the cell envelope of Halobacterium salinarium. *Journal of Biological Chemistry*.

[3] Young NM, Brisson JR, Kelly J (2002). Structure of the N-linked glycan present on multiple glycoproteins in the gram-negative bacterium, Campylobacter jejuni. *Journal of Biological Chemistry*.

[4] Wacker M, Linton D, Hitchen PG (2002). N-linked glycosylation in Campylobacter jejuni and its functional transfer into E. coli. *Science*.

[5] Calo D, Kaminski L, Eichler J (2010). Protein glycosylation in Archaea: sweet and extreme. *Glycobiology*.

[6] Hug I, Feldman MF (2011). Analogies and homologies in lipopolysaccharide and glycoprotein biosynthesis in bacteria. *Glycobiology*.

[7] Varki A, Cummings RD, Esko JD (2009). *Essentials of Glycobiology*.

[8] Taylor MT, Drickamer K (2006). *Introduction to Glycobiology*.

[9] Kelleher DJ, Banerjee S, Cura AJ, Samuelson J, Gilmore R (2007). Dolichol-linked oligosaccharide selection by the oligosaccharyltransferase in protist and fungal organisms. *Journal of Cell Biology*.

[10] Izquierdo L, Schulz BL, Rodrigues JA (2009). Distinct donor and acceptor specificities of Trypanosoma brucei oligosaccharyltransferases. *The EMBO Journal*.

[11] Sanyal S, Menon AK (2010). Stereoselective transbilayer translocation of mannosyl phosphoryl dolichol by an endoplasmic reticulum flippase. *Proceedings of the National Academy of Sciences of the United States of America*.

[12] Helenius J, Ng DTW, Marolda CL, Walter P, Valvano MA, Aebi M (2002). Translocation of lipid-linked oligosaccharides across the ER membrane requires Rft1 protein. *Nature*.

[13] Raetz CRH, Reynolds CM, Trent MS, Bishop RE (2007). Lipid a modification systems in gram-negative bacteria. *Annual Review of Biochemistry*.

[14] Alaimo C, Catrein I, Morf L (2006). Two distinct but interchangeable mechanisms for flipping of lipid-linked oligosaccharides. *The EMBO Journal*.

[15] Cuthbertson L, Kos V, Whitfield C (2010). ABC transporters involved in export of cell surface glycoconjugates. *Microbiology and Molecular Biology Reviews*.

[16] Abu-Qarn M, Eichler J (2006). Protein N-glycosylation in Archaea: defining Haloferax volcanii genes involved in S-layer glycoprotein glycosylation. *Molecular Microbiology*.

[17] Abu-Qarn M, Yurist-Doutsch S, Giordano A (2007). Haloferax volcanii AglB and AglD are involved in N-glycosylation of the S-layer glycoprotein and proper assembly of the surface layer. *Journal of Molecular Biology*.

[18] Jervis AJ, Langdon R, Hitchen P (2010). Characterization of N-linked protein glycosylation in Helicobacter pullorum. *Journal of Bacteriology*.

[19] Hitchen PG, Twigger K, Valiente E, Langdon RH, Wren BW, Dell A (2010). Glycoproteomics: a powerful tool for characterizing the diverse glycoforms of bacterial pilins and flagellins. *Biochemical Society Transactions*.

[20] Lennarz WJ (2007). Studies on oligosaccharyl transferase in yeast. *Acta Biochimica Polonica*.

[21] Kelleher DJ, Gilmore R (2006). An evolving view of the eukaryotic oligosaccharyltransferase. *Glycobiology*.

[22] Schulz BL, Aebi M (2009). Analysis of glycosylation site occupancy reveals a role for Ost3p and Ost6p in site-specific N-glycosylation efficiency. *Molecular and Cellular Proteomics*.

[23] Nasab FP, Schulz BL, Gamarro F, Parodi AJ, Aebi M (2008). All in one: leishmania major STT3 proteins substitute for the whole oligosaccharyltransferase complex in Saccharomyces cerevisiae. *Molecular Biology of the Cell*.

[24] Hese K, Otto C, Routier FH, Lehle L (2009). The yeast oligosaccharyltransferase complex can be replaced by STT3 from Leishmania major. *Glycobiology*.

[25] Ruiz-Canada C, Kelleher DJ, Gilmore R (2009). Cotranslational and posttranslational N-glycosylation of polypeptides by distinct mammalian OST isoforms. *Cell*.

[26] Feldman MF, Wacker M, Hernandez M (2005). Engineering N-linked protein glycosylation with diverse O antigen lipopolysaccharide structures in Escherichia coli. *Proceedings of the National Academy of Sciences of the United States of America*.

[27] Peyfoon E, Meyer B, Hitchen PG The S-layer glycoprotein of the crenarchaeote Sulfolobus acidocaldarius is glycosylated at multiple sites with chitobiose-linked N-glycans.

[28] Kowarik M, Young NM, Numao S (2006). Definition of the bacterial N-glycosylation site consensus sequence. *The EMBO Journal*.

[29] Zielinska DF, Gnad F, Wiśniewski JR, Mann M (2010). Precision mapping of an in vivo N-glycoproteome reveals rigid topological and sequence constraints. *Cell*.

[30] Claus H, Akça E, Debaerdemaeker T (2005). Molecular organization of selected prokaryotic S-layer proteins. *Canadian Journal of Microbiology*.

[31] Messner P, Steiner K, Zarschler K, Schäffer C (2008). S-layer nanoglycobiology of bacteria. *Carbohydrate Research*.

[32] Grass S, Buscher AZ, Swords WE (2003). The Haemophilus influenzae HMW1 adhesin is glycosylated in a process that requires HMW1C and phosphoglucomutase, an enzyme involved in lipooligosaccharide biosynthesis. *Molecular Microbiology*.

[33] Gross J, Grass S, Davis AE, Gilmore-Erdmann P, Townsend RR, St Geme JW (2008). The Haemophilus influenzae HMW1 adhesin is a glycoprotein with an unusual N-linked carbohydrate modification. *Journal of Biological Chemistry*.

[34] Grass S, Lichti CF, Townsend RR, Gross J, St Geme JW (2010). The haemophilus influenzae HMW1c protein is a glycosyltransferase that transfers hexose residues to asparagine sites in the HMW1 adhesin. *PLoS Pathogens*.

[35] Schreiner R, Schnabel E, Wieland F (1994). Novel N-glycosylation in eukaryotes: laminin contains the linkage unit *β*-glucosylasparagine. *Journal of Cell Biology*.

[36] Stimson E, Virji M, Makepeace K (1995). Meningococcal pilin: a glycoprotein substituted with digalactosyl 2,4-diacetamido-2,4,6-trideoxyhexose. *Molecular Microbiology*.

[37] Hegge FT, Hitchen PG, Aas FE (2004). Unique modifications with phosphocholine and phosphoethanolamine define alternate antigenic forms of Neisseria gonorrhoeae type IV pili. *Proceedings of the National Academy of Sciences of the United States of America*.

[38] Power PM, Seib KL, Jennings MP (2006). Pilin glycosylation in Neisseria meningitidis occurs by a similar pathway to wzy-dependent O-antigen biosynthesis in Escherichia coli. *Biochemical and Biophysical Research Communications*.

[39] Faridmoayer A, Fentabil MA, Mills DC, Klassen JS, Feldman MF (2007). Functional characterization of bacterial oligosaccharyltransferases involved in O-linked protein glycosylation. *Journal of Bacteriology*.

[40] Qutyan M, Paliotti M, Castric P (2007). PilO of Pseudomonas aeruginosa 1244: subcellular location and domain assignment. *Molecular Microbiology*.

[41] Smedley JG, Jewell E, Roguskie J (2005). Influence of pilin glycosylation on Pseudomonas aeruginosa 1244 pilus function. *Infection and Immunity*.

[42] Vik A, Aas FE, Anonsen JH (2009). Broad spectrum O-linked protein glycosylation in the human pathogen Neisseria gonorrhoeae. *Proceedings of the National Academy of Sciences of the United States of America*.

[43] Ku SC, Schulz BL, Power PM, Jennings MP (2009). The pilin O-glycosylation pathway of pathogenic Neisseria is a general system that glycosylates AniA, an outer membrane nitrite reductase. *Biochemical and Biophysical Research Communications*.

[44] Faridmoayer A, Fentabil MA, Haurat MF (2008). Extreme substrate promiscuity of the Neisseria oligosaccharyl transferase involved in protein O-glycosylation. *Journal of Biological Chemistry*.

[45] Coyne MJ, Reinap B, Lee MM, Comstock LE (2005). Human symbionts use a host-like pathway for surface fucosylation. *Science*.

[46] Fletcher CM, Coyne MJ, Villa OF, Chatzidaki-Livanis M, Comstock LE (2009). A general O-glycosylation system important to the physiology of a major human intestinal symbiont. *Cell*.

[47] Zeituni AE, McCaig W, Scisci E, Thanassi DG, Cutler CW (2010). The native 67-kilodalton minor fimbria of Porphyromonas gingivalis is a novel glycoprotein with DC-SIGN-targeting motifs. *Journal of Bacteriology*.

[48] Sumper M, Berg E, Mengele R, Strobel I (1990). Primary structure and glycosylation of the S-layer protein of Haloferax volcanii. *Journal of Bacteriology*.

[49] Schäffer C, Graninger M, Messner P (2001). Prokaryotic glycosylation. *Proteomics*.

[50] Abu-Qarn M, Eichler J, Sharon N (2008). Not just for Eukarya anymore: protein glycosylation in Bacteria and Archaea. *Current Opinion in Structural Biology*.

[51] Wu H, Zeng M, Fives-Taylor P (2007). The glycan moieties and the N-terminal polypeptide backbone of a fimbria-associated adhesin, Fap1, play distinct roles in the biofilm development of Streptococcus parasanguinis. *Infection and Immunity*.

[52] Peng Z, Fives-Taylor P, Ruiz T (2008). Identification of critical residues in Gap3 of Streptococcus parasanguinis involved in Fap1 glycosylation, fimbrial formation and in vitro adhesion. *BMC Microbiology*.

[53] Zhou M, Wu H (2009). Glycosylation and biogenesis of a family of serine-rich bacterial adhesins. *Microbiology*.

[54] Ramboarina S, Garnett JA, Zhou M (2010). Structural insights into serine-rich fimbriae from gram-positive bacteria. *Journal of Biological Chemistry*.

[55] Bensing BA, Gibson BW, Sullam PM (2004). The Streptococcus gordonii platelet binding protein GspB undergoes glycosylation independently of export. *Journal of Bacteriology*.

[56] van Sorge NM, Quach D, Gurney MA, Sullam PM, Nizet V, Doran KS (2009). The group B streptococcal serine-rich repeat 1 glycoprotein mediates penetration of the blood-brain barrier. *Journal of Infectious Diseases*.

[57] Shivshankar P, Sanchez C, Rose LF, Orihuela CJ (2009). The Streptococcus pneumoniae adhesin PsrP binds to Keratin 10 on lung cells. *Molecular Microbiology*.

[58] Bu SU, Li Y, Zhou M (2008). Interaction between two putative glycosyltransferases is required for glycosylation of a serine-rich streptococcal adhesin. *Journal of Bacteriology*.

[59] Wu R, Zhou M, Wu H (2010). Purification and characterization of an active N-acetylglucosaminyltransferase enzyme complex from Streptococci. *Applied and Environmental Microbiology*.

[60] Zeidan Q, Hart GW (2010). The intersections between O-GlcNAcylation and phosphorylation: implications for multiple signaling pathways. *Journal of Cell Science*.

[61] Zhou M, Zhu F, Dong S, Pritchard DG, Wu H (2010). A novel glucosyltransferase is required for glycosylation of a serine-rich adhesin and biofilm formation by Streptococcus parasanguinis. *Journal of Biological Chemistry*.

[62] Lommel M, Strahl S (2009). Protein O-mannosylation: conserved from bacteria to humans. *Glycobiology*.

[63] Endo T (1999). O-Mannosyl glycans in mammals. *Biochimica et Biophysica Acta*.

[64] Nakamura N, Lyalin D, Panin VM (2010). Protein O-mannosylation in animal development and physiology: from human disorders to Drosophila phenotypes. *Seminars in Cell and Developmental Biology*.

[65] Espitia C, Servín-González L, Mancilla R (2010). New insights into protein O-mannosylation in actinomycetes. *Molecular BioSystems*.

[66] Dobos KM, Khoo KH, Swiderek KM, Brennan PJ, Belisle JT (1996). Definition of the full extent of glycosylation of the 45-kilodalton glycoprotein of Mycobacterium tuberculosis. *Journal of Bacteriology*.

[67] Sartain MJ, Belisle JT (2009). N-Terminal clustering of the O-glycosylation sites in the Mycobacterium tuberculosis lipoprotein SodC. *Glycobiology*.

[68] Michell SL, Whelan AO, Wheeler PR (2003). The MPB83 antigen from Mycobacterium bovis contains O-linked mannose and (1 → 3)-mannobiose moieties. *Journal of Biological Chemistry*.

[69] Mahne M, Tauch A, Pühler A, Kalinowski J (2006). The Corynebacterium glutamicum gene pmt encoding a glycosyltransferase related to eukaryotic protein-O-mannosyltransferases is essential for glycosylation of the resuscitation promoting factor (Rpf2) and other secreted proteins. *FEMS Microbiology Letters*.

[70] Wehmeier S, Varghese AS, Gurcha SS (2009). Glycosylation of the phosphate binding protein, PstS, in Streptomyces coelicolor by a pathway that resembles protein O-mannosylation in eukaryotes. *Molecular Microbiology*.

[71] Benz I, Schmidt MA (2001). Glycosylation with heptose residues mediated by the aah gene product is essential for adherence of the AIDA-I adhesin. *Molecular Microbiology*.

[72] Sherlock O, Dobrindt U, Jensen JB, Vejborg RM, Klemm P (2006). Glycosylation of the self-recognizing Escherichia coli Ag43 autotransporter protein. *Journal of Bacteriology*.

[73] Reidl S, Lehmann A, Schiller R, Salam Khan A, Dobrindt U (2009). Impact of O-glycosylation on the molecular and cellular adhesion properties of the Escherichia coli autotransporter protein Ag43. *International Journal of Medical Microbiology*.

[74] Knudsen SK, Stensballe A, Franzmann M, Westergaard UB, Otzen DE (2008). Effect of glycosylation on the extracellular domain of the Ag43 bacterial autotransporter: enhanced stability and reduced cellular aggregation. *Biochemical Journal*.

[75] Hitchen PG, Twigger K, Valiente E, Langdon RH, Wren BW, Dell A (2010). Glycoproteomics: a powerful tool for characterizing the diverse glycoforms of bacterial pilins and flagellins. *Biochemical Society Transactions*.

[76] Logan SM (2006). Flagellar glycosylation—a new component of the motility repertoire?. *Microbiology*.

[77] Miller WL, Matewish MJ, McNally DJ (2008). Flagellin glycosylation in Pseudomonas aeruginosa PAK requires the O-antigen biosynthesis enzyme WbpO. *Journal of Biological Chemistry*.

[78] Twine SM, Reid CW, Aubry A (2009). Motility and flagellar glycosylation in Clostridium difficile. *Journal of Bacteriology*.

[79] Julenius K, Mølgaard A, Gupta R, Brunak S (2005). Prediction, conservation analysis, and structural characterization of mammalian mucin-type O-glycosylation sites. *Glycobiology*.

[80] Herrmann JL, Delahay R, Gallagher A, Robertson B, Young D (2000). Analysis of post-translational modification of mycobacterial proteins using a cassette expression system. *FEBS Letters*.

